# High glucose-induced PLCG1 histone acetylation to promote ferroptosis by LAMP2A/HSPA8 in a diabetic nephropathy model

**DOI:** 10.3389/fphar.2025.1640721

**Published:** 2026-01-14

**Authors:** Jun Ge, Zhenzhen Wang, Ting Xu, Ruifeng Jiang, Xuefeng Zhang

**Affiliations:** 1 Department of Nephrology, Yantai Affiliated Hospital of Binzhou Medical University, Yantai, China; 2 Department of Pharmacy, Yantai Affiliated Hospital of Binzhou Medical University, Yantai, China

**Keywords:** diabetic nephropathy, PLCG1, histone acetylation, ferroptosis, treatment

## Abstract

Diabetic nephropathy (DN) is one of the most prevalent microvascular complications of diabetes mellitus. In the present study, the effects of PLCG1 DN, as well as its underlying molecular mechanisms associated with ferroptosis, were investigated. Single-cell RNA sequencing data and bioinformatic analyses were employed to support these experimental findings. For *in vivo* experiments, a DN model was established in C57BL/6 mice via streptozotocin injection. For *in vitro* investigations, NRK-52E cells were exposed to 20 mmol/L d-glucose to induce a DN-like cellular phenotype. PLCG1 mRNA expression levels were upregulated in DN patients, compared with the normal group. Elevated serum PLCG1 mRNA expression in DN patients correlated with increased urinary creatinine (Cre), blood urea nitrogen (Bun), and 24 h urinary microalbuminuria (mAlb) levels. The mRNA and protein expression levels of PLCG1 m in tissues were significantly upregulated in the mouse DN model and high glucose-induced NRK-52E. Single-cell analysis was performed to detect PLCG1 expression in renal cells of the DN model. Additionally, high glucose exposure induced PLCG1 histone acetylation in the DN model. Sh-PLCG1 alleviated DN progression and reduced oxidative stress in the mouse model. Mechanistically, PLCG1 increased mitochondria-dependent ferroptosis in the DN model. PLCG1 is interlinked with LAMP2A and facilitates the ubiquitination of LAMP2A. Specifically, PLCG1 upregulation enhanced K48-linked ubiquitination of LAMP2A protein in high glucose-induced NRK-52E cells. Ultimately, PLCG1 inhibited the LAMP2A/HSPA8 signaling pathway in the DN model. Our study identifies PLCG1 as a novel regulatory target that inhibits the LAMP2A/HSPA8 signaling pathway. This inhibition promotes mitochondrial oxidative stress, which in turn increases cellular ferroptosis and accelerates the progression of DN. Importantly, PLCG1 holds promise as a critical clinical biomarker for diagnosing DN. It may serve as a potential therapeutic target to mitigate glucose-induced ferroptosis, with implications for the management of not only DN but also other diabetes-related complications.

## Introduction

Diabetes mellitus is a prevalent metabolic disorder that poses a significant threat to human health (Chen et al., 2025). Prolonged metabolic disturbances and persistent hyperglycemia can progressively exacerbate damage to multiple organs and cell types in affected patients, including the kidneys, heart, podocytes, retina, cerebrovascular system, and nervous system, thereby contributing to the development of numerous complications (Gu et al., 2025). Among its chronic complications, diabetic retinopathy and diabetic nephropathy (DN) are the most prominent (Lin et al., 2025). Currently, China has the largest population of diabetes patients worldwide (Lin et al., 2025). Diabetic nephropathy (DN) is a common microvascular complication of diabetes mellitus, characterized clinically by persistent albuminuria and/or a gradual decline in glomerular filtration rate (Zhang et al., 2024). To date, the number of patients with chronic kidney disease secondary to diabetes has reached 24 million, and the risk of diabetes progressing to DN can be as high as 40% (Elhenawy et al., 2025).

Oxidative stress, inflammation, autophagy, and epigenetic regulation have emerged as key pathological mechanisms in current research on DN (Jin et al., 2023). These mechanisms are closely interrelated (Jin et al., 2023): through complex signal transduction pathways, they interact reciprocally and collectively drive the development and progression of DN (Chen et al., 2022).

Regulating these signaling molecules may be a way to treat diabetic nephropathy. Diabetic nephropathy encompasses all types of renal injury occurring in patients with diabetes (Ma et al., 2021). At present, there remains a lack of effective treatment methods for diabetic nephropathy (Samsu, 2021). Clinically, the main approach is to control blood glucose, blood pressure, and blood lipids to prevent the progression of diabetic nephropathy. Traditional Chinese medicine has a unique theoretical foundation and extensive clinical experience in treating diabetic nephropathy (Ma et al., 2021). A large body of evidence has confirmed that numerous TCM monomers and compound preparations can delay DN progression by targeting autophagy as a therapeutic mediator (Xu et al., 2023).

The pathogenesis of DN has not been fully elucidated, with multiple mechanisms involved, including oxidative stress, inflammatory response, accumulation of advanced glycation end products, endoplasmic reticulum stress, autophagy, and pyroptosis (Yang et al., 2025). Ferroptosis is an iron-dependent form of programmed cell death, typically accompanied by iron overload and lipid peroxidation (Yuan et al., 2025). Currently, it is widely recognized that iron overload, free radical generation, fatty acid supply, and lipid peroxidation are the key factors that trigger ferroptosis (Wang et al., 2023). Previous studies have demonstrated that renal iron overload is a common phenomenon in diabetic animal models; furthermore, serum creatinine and urinary protein are positively correlated with renal iron and ferritin levels (Wang et al., 2025; Wu B. et al., 2025a). Ferroptosis in DN has been reported (Song and Yu, 2025). Therefore, targeted inhibition of ferroptosis is expected to become a novel therapeutic direction for DN.

Some studies have demonstrated that histone acetylation modification mediated by histone deacetylases (HDACs) plays a crucial role in the occurrence and development of DN (Khan et al., 2016; Wang et al., 2014). Specifically, the application of HDAC inhibitors has been shown to delay or reverse the progression of DN (Li et al., 2016). Among the HDAC family members, HDAC4 can inhibit podocyte autophagy and exacerbate inflammatory responses during the pathogenesis of DN (Hadden and Advani, 2018).

The PLCG1 gene is located on chromosome 20q12-13.1 and encodes phospholipase Cγ (Cheng et al., 2025). By mediating the inositol signaling pathway, PLCG1 participates in cellular processes such as cell differentiation and proliferation, and it has been implicated in the pathogenesis of hematological malignancies (Cheng et al., 2025). For instance, studies have identified a single allele deletion of PLCG1 in myelodysplastic syndrome (MDS) patients, suggesting that this genetic alteration may serve as a novel molecular marker for the diagnosis and treatment of MDS (Li et al., 2023). Additionally, PLCG1 can activate the expression of Janus kinase 2 and its downstream molecule, the erythropoietin receptor, and promote the proliferation and differentiation of red blood cells (Li et al., 2022). Consistently, the differentiation and maturation of the erythroid lineage are impaired in PLCG1 knockout mice (Schnoeder et al., 2022). In the present study, we investigated the effects of PLCG1 on DN and its underlying molecular mechanisms related to ferroptosis.

## Materials and methods

### Single-cell RNA sequencing data and bioinformatic analyses

Single-cell RNA sequencing data and bioinformatic analyses were performed using GSE195460 or GSE255028.

### Animal experiments

The animal studies were authorized by the Animal Ethics Review Committee of the Yijishan Hospital of Wannan Medical College. All animal experiments were strictly implemented in compliance with the NIH Guide for the Care and Use of Laboratory Animals. C57BL/6 mice (male; age, 5–6 weeks; weight, 18–20 g) were housed in a specific pathogen-free (SPF) environment and randomly assigned to different groups.

DN mice were fed with a high-fat diet (HFD) for 12 weeks and then injected with STZ (30 mg/kg of streptozotocin, Sigma-Aldrich) i.p. for 7 consecutive days. Blood glucose levels were measured using 16.7 mmol/L after 1 week of the final injection. Negative (10^9^ PFU/mL, 100 µl/3 days) or sh-PLCG1 lentivirus (10^9^ PFU/mL, 100 µl/3 days) was injected into the mice via the tail vein for 6 weeks. Mice were killed under anesthesia, and kidneys and serum were taken for analysis.

Kidney tissue samples were fixed in 4% paraformaldehyde, paraffin-embedded, and then sectioned into 5-μm slices for HE, Masson staining, and periodic acid–Schiff (PAS) staining. Kidney tissue samples were observed using a fluorescence microscope (Zeiss Axio Observer A1, Germany).

### ELISA and cell viability assay

Malondialdehyde activity (MDA) (A003-1-2), superoxide dismutase (SOD) (A001-3-2), glutathione (GSH) (A006-2-1), and glutathione peroxidase (GSH-PX) (A005-1-2) testing was performed as described in a previous study (Pu et al., 2024). Cell viability was determined using a CCK-8 assay (C0037, Beyotime Biotechnology), as described in a previous study (Pu et al., 2023). Absorbance was measured on the Synergy H1 Microplate Reader (BioTek, Winooski).

### Histological, immunohistochemical, and immunofluorescence analyses and electron microscopy

For immunohistochemical and immunofluorescence analyses, mouse kidney tissue samples were fixed in 4% paraformaldehyde and stained with hematoxylin and eosin (HE), as described previously (Pu et al., 2022). Tissue samples were observed under a fluorescence microscope (Zeiss Axio Observer A1, Germany) and a transmission electron microscope (80 kV) (Hitachi H-7650, Tokyo, Japan), as described in a previous study (Pu et al., 2023). PLCG1 (1:100, Abcam) and LAMP2A (1:100, Abcam) were used for immunohistochemical and immunofluorescence analyses.

### Real-time PCR

Total RNAs were isolated with RNA isolator total RNA extraction reagent (Takara), and cDNA was synthesized using PrimeScript RT Master Mix (Takara). qPCR was performed with the ABI Prism 7500 sequence detection system according to the Prime-Script™ RT detection kit. Relative levels of the sample mRNA expression were calculated and expressed as 2^−△△Ct^. The primer showed that PLCG1: upstream primer: 5′-GTA​CTG​CAT​CGA​GAC​CGG​AG-3′; downstream primer: 5′-GGG​CTT​TGA​CTG​CAC​ACT​TG-3′; GAPDH: upstream primer: 5′-CTG​GGC​TAC​ACT​GAG​CAC​C-3′; downstream primer: 5′-AAG​TGG​TCG​TTG​AGG​GCA​ATG-3′. The PCR procedure with the following reaction conditions: at 95 °C for 2 min, followed by 40 cycles of 95 °C for 15 s and 60 °C for 1 min.

### Western blotting analysis and immunofluorescence

PLCG1 (ab76031, Abcam), LAMP2A (ab240018, Abcam), HSPA8 (ab51052, Abcam), GPX4 (ab125066, Abcam), β-actin (1:10000, AC028, ABclonal, Inc.) and anti-rabbit IgG (1:5,000, GB23303, ServiceBio) were used in this study. Protein was measured using a BeyoECL Plus kit (P0018S) and analyzed using Image Lab 3.0 (Bio-Rad Laboratories, Inc.). All results were repeated three times. Western blotting analysis and immunofluorescence were executed according to Pu et al. (2023).

### *In vitro* model

NRK-52E cells were maintained in DMEM (Gibco) with 10% FBS (Gibco) under a humidified 5% (v/v) CO_2_ atmosphere at 37 °C according to Zhang et al. (2024). NRK-52E was stimulated with 20 mmol/L d-glucose for the DN model. Next, the transfections were performed using Lipofectamine 2000 (Thermo Fisher Scientific). After 48 h of transfection, NRK-52E was stimulated with 20 mmol/L D-glucose.

### Microscale thermophoresis (MST), thermal shift assay (TSA), and cellular thermal shift assay (CETSA)

MST, TSA, and CETSA were executed as described previously (Zhang et al., 2024). A 0.10 mg/mL aliquot of WT WWP2 protein was used with or without 0.30 mmol/L abietic acid in PBS. A 20 μL aliquot of the supernatant was used for Western blotting. CETSA and thermal stability were performed using GraphPad Prism software (GraphPad, San Diego, CA, United States).

### Co-immunoprecipitation assay, the m^6^A quantification, luciferase reporter assay, and m^6^A RNA immunoprecipitation (MeRIP) assay

These experiments were performed as previously described (Yuan et al., 2024). The immunoprecipitated RNA was digested, purified, and further analyzed by qPCR. m^6^A mRNA levels were colorimetrically measured by ELISA assay with an EpiQuik m^6^A RNA Methylation Quantification kit, and Renilla luciferase activities were determined using a Dual-Luciferase Assay kit (Promega). Poly(A) + mRNA was isolated using the Dynabeads mRNA Direct Purification Kit (B518710, Sangon Biotech (Shanghai) Co., Ltd.). Purified RNA (2 μg) was used in a Magna MeRIP m^6^A Kit (17-10499, Millipore) for m^6^A-containing mRNA enrichment.

### Statistical analysis

P < 0.05 was considered significant and evaluated using Student’s t-test or one-way analysis of variance (ANOVA) followed by Tukey’s post-hoc test. Data were expressed as mean ± standard deviation (SD).

## Results

### PLCG1 expression levels were upregulated in a DN model

**FIGURE 1:**
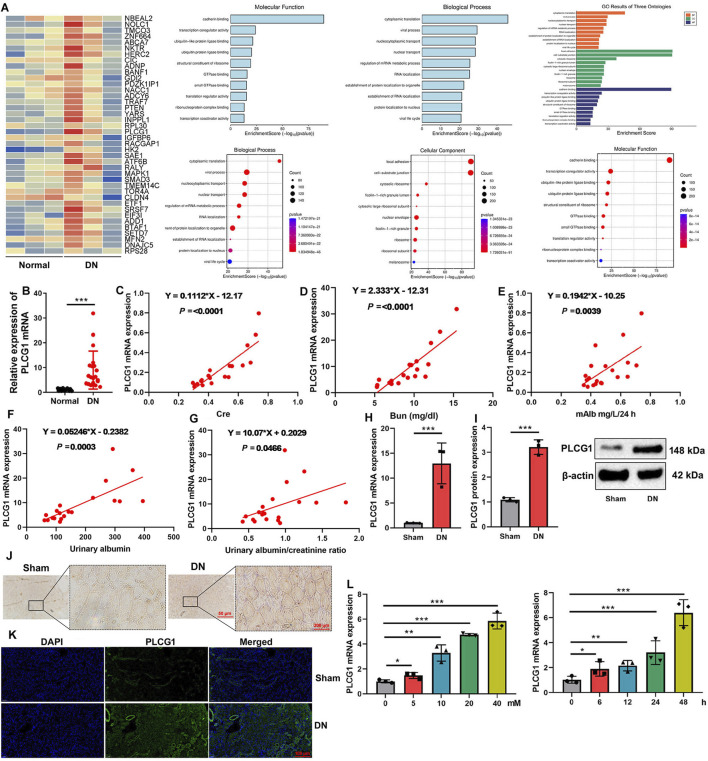
PLCG1 expression levels in a model of the DN heat map **(A)**, serum PLCG1 mRNA expression **(B)** in DN patients correlated with increased Cre **(C)**, Bun **(D)**, 24 h mAlb levels **(E)**, urinary albumin **(F),** and urinary albumin/creatinine ratio **(G)** in patients with DN; PLCG1 mRNA and protein expression **(H,I)**, PLCG1 expression levels in lung tissue of DN mice (immunohistochemistry, **(J)** and immunofluorescence, **(K)**) in DN mice; PLCG1 mRNA expression levels **(L)** in high glucose-induced NRK-52E. ^*^, P < 0.05; ^**^, P < 0.01; ^***^, P < 0.001

In this study, we initially employed gene chip technology to investigate genes associated with the onset and progression of DN. Analysis revealed a significant upregulation of PLCG1 expression in DN patients (Figure 1A). Consistent with this finding, PCR results demonstrated elevated levels of PLCG1 mRNA in the blood of DN patients (Figure 1B). Elevated serum PLCG1 mRNA expression in DN patients correlated with increased urinary Cre, Bun, 24 h mAlb levels, urinary albumin, and urinary albumin/creatinine ratio (Figures 1C–G).

PLCG1 mRNA and protein expression levels in kidney tissue were upregulated in a mouse DN model (Figures 1H,I). Immunohistochemistry and immunofluorescence assays also demonstrated that PLCG1 expression levels in the kidney tissue of the DN mice model were upregulated (Figures 1J,K). Meanwhile, PLCG1 dose- and time-dependently increased mRNA expression levels in high glucose-induced NRK-52E (Figure 1L). As a result, PLCG1 expression levels in the DN model were upregulated.

### Single-cell analysis of PLCG1 expression levels in renal cells of the DN model

**FIGURE 2:**
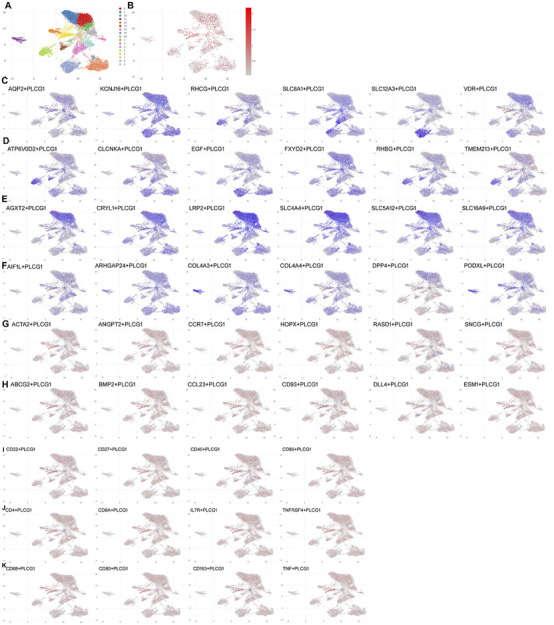
Single-cell analysis of PLCG1 expression levels in renal cells of the DN model. Single-cell sequencing data for PLCG1 expression **(A,B)**. Distal tubule cells (AQP2/KCNJ16/RHCG/SLC8A1/SLC12A3/VDR **(C)**), intercalated cells (ATP6V0D2/CLCNKA/EGF/FXYD2/RHBG/TMEM213 **(D)**), proximal tubule cells (AGXT2/CRYL1/LRP2/SLC4A4/SLC5A12/SLC16A9 **(E)**), podocytes (AIF1L/ARHGAP24/COL4A3/COL4A4/DPP4/PODXL **(F)**), mesangial cells (ACTA2/ANGPT2/CCR7/HOPX/RASD1/SNCG **(G)**, endothelial cells (ABCG2/BMP2/CCL23/CD93/DLL4/ESM1 **(H)**, B cells (CD22/CD27/CD40/CD86 **(I)**), T cells (CD4/CD8A/IL7R/TNFRSF4 **(J)**), and macrophage cells (CD68/CD80/CD163/TNF **(K)**) in DN patients.

We further investigated the mechanistic role of PLCG1 in a DN model by employing single-cell RNA sequencing. Subsequent analysis confirmed a significant upregulation of PLCG1 expression in renal cells from DN patients (Figures 2A,B). PLCG1 was found to be expressed in the distal tubule cells (AQP2/KCNJ16/RHCG/SLC8A1/SLC12A3/VDR), intercalated cells (ATP6V0D2/CLCNKA/EGF/FXYD2/RHBG/TMEM213), proximal tubule cells (AGXT2/CRYL1/LRP2/SLC4A4/SLC5A12/SLC16A9), and podocytes (AIF1L/ARHGAP24/COL4A3/COL4A4/DPP4/PODXL) of DN patients (Figures 2C–F). However, PLCG1 was not manifested in mesangial cells (ACTA2/ANGPT2/CCR7/HOPX/RASD1/SNCG), endothelial cells (ABCG2/BMP2/CCL23/CD93/DLL4/ESM1), B cells (CD22/CD27/CD40/CD86), T cells (CD4/CD8A/IL7R/TNFRSF4), or macrophage cells (CD68/CD80/CD163/TNF) of DN patients (Figures 2G–K). Consequently, PLCG1 expression levels are increased in the renal cells of the DN model.

### High glucose-induced PLCG1 histone acetylation in a DN model

**FIGURE 3:**
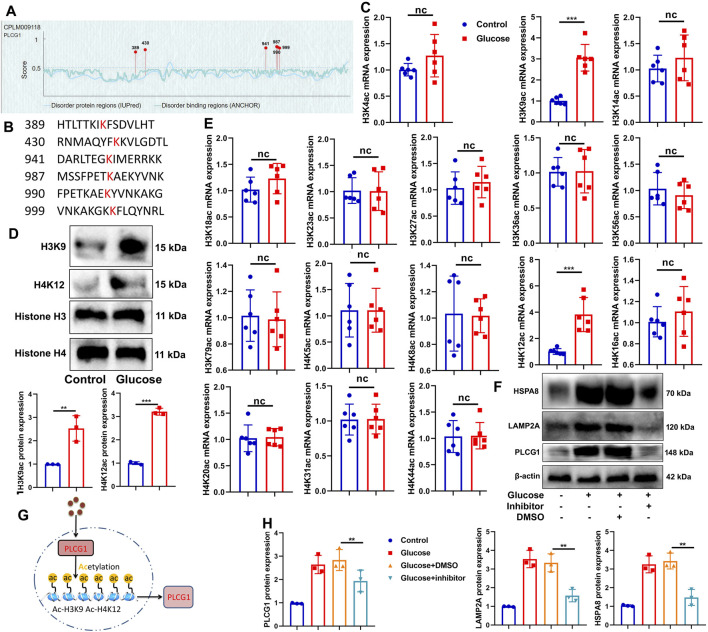
High glucose induced PLCG1 histone acetylation in the histone acetylation sites in the DN model **(A,B)**, H3K9ac/H4K12ac mRNA and protein expression **(C–E)** in high glucose-induced NRK-52E cells; PLCG1/LAMP2A/HSPA8 protein expression **(F,H)** in high glucose-induced NRK-52E cells by histone acetylation inhibitor (trichostatin A, 1.8 nM); experimental description **(G)**. ^**^, P < 0.01; ^***^, P < 0.001.

We next sought to determine the cause of aberrant PLCG1 expression in DN. Bioinformatic analysis revealed that PLCG1 contains six potential histone acetylation sites (Figures 3A,B). Experimental studies demonstrated that high glucose upregulates both mRNA and protein expression of H3K9ac/H4K12ac in NRK-52E cells (Figures 3C–E), while also inducing the protein expression of PLCG1/LAMP2A/HSPA8 (Figure 3F). Crucially, treatment with the histone acetylation inhibitor trichostatin A (1.8 nM) suppressed the high glucose-induced elevation of PLCG1, LAMP2A, and HSPA8 protein levels (Figures 3H,H). These findings collectively indicate that high glucose promotes histone acetylation of PLCG1 in the DN model (Figure 3G).

### Sh-PLCG1 reduced DN and oxidative stress in the mouse model

**FIGURE 4:**
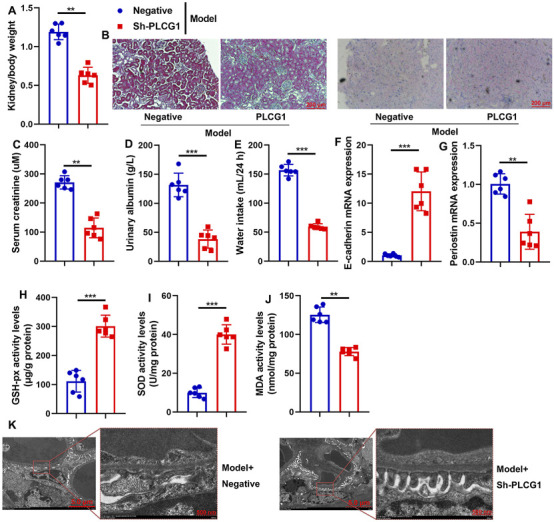
Sh-PLCG1 reduced DN and oxidative stress in mice model kidney/body weight **(A)**, renal fibrosis (HE and PAS staining **(B)**), serum creatinine **(C)**, urinary axzlbumin levels **(D)**, water intake **(E)**, E-cadherin and periostin mRNA expression **(F,G)**, GSH-PX, SOD, and MDA activity levels **(H–J)**, and podocyte ultrastructure changes (electron microscope, **(K)**) in the DN mice model. ^**^, P < 0.01; ^***^, P < 0.001.

Subsequently, this study examined the therapeutic effects of sh-PLCG1 on renal injury in a mouse DN model using the sh-PLCG1 virus. Treatment with sh-PLCG1 virus significantly ameliorated renal injury, as evidenced by reduced kidney-to-body weight ratios and serum creatinine levels, decreased urinary albumin excretion and water intake, and improved renal histology with attenuated fibrosis (HE and PAS staining) (Figures 4A–E). Further analysis demonstrated that sh-PLCG1 upregulated E-cadherin mRNA expression while suppressing periostin mRNA levels in renal tissue. It also markedly reduced lipid peroxidation (MDA activity) and enhanced antioxidant capacity (GSH-Px and SOD activities) (Figures 4F–J). Electron microscopy confirmed that PLCG1 knockdown improved podocyte ultrastructural alterations in DN mice (Figure 4K). Collectively, these results indicate that sh-PLCG1 alleviates the progression of diabetic nephropathy and oxidative stress in the experimental model.

### PLCG1 increased mitochondrial ROS accumulation in the DN model

**FIGURE 5:**
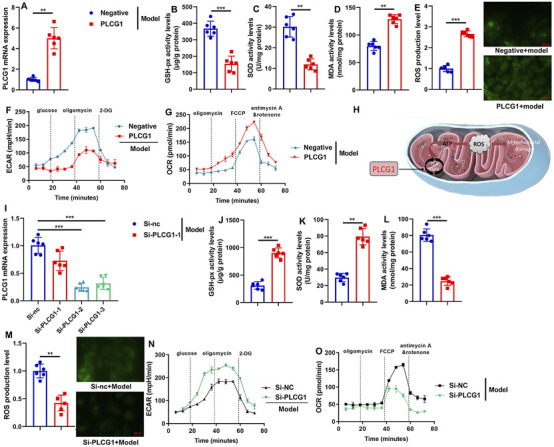
PLCG1 mRNA expression levels **(A)**, GSH-PX, SOD, MDA and ROS activity levels **(B–E)**, ECAR levels **(F)**, OCR levels **(G)** in vitro model by PLCG1 plasmid; Experimental Description **(H)**, PLCG1 mRNA expression levels **(I)**, GSH-PX, SOD, MDA and ROS activity levels **(J–M)**, ECAR levels **(N)**, OCR levels **(O)** *in vitro* model by si-PLCG1 plasmid; **, P < 0.01; ***, P < 0.001.

We next evaluated the impact of PLCG1 on mitochondrial reactive oxygen species (ROS) accumulation in a DN model. Overexpression of PLCG1 via plasmid transfection significantly upregulated PLCG1 mRNA expression, decreased GSH-Px and SOD activities, and elevated MDA levels and ROS production in the *in vitro* DN model (Figures 5A–E). Furthermore, PLCG1 overexpression suppressed extracellular acidification rate (ECAR) and increased oxygen consumption rate (OCR) (Figures 5F,G), suggesting that PLCG1 activates substantial mitochondrial ROS generation by promoting the tricarboxylic acid cycle to accelerate ATP production (Figure 5H).

Conversely, siRNA-mediated PLCG1 knockdown reduced PLCG1 mRNA expression, increased GSH-Px and SOD activities, and lowered both MDA and ROS levels (Figures 5I–O). These results collectively demonstrate that PLCG1 drives mitochondrial ROS accumulation in diabetic nephropathy.

### PLCG1 increased mitochondria-dependent ferroptosis in the DN model

**FIGURE 6:**
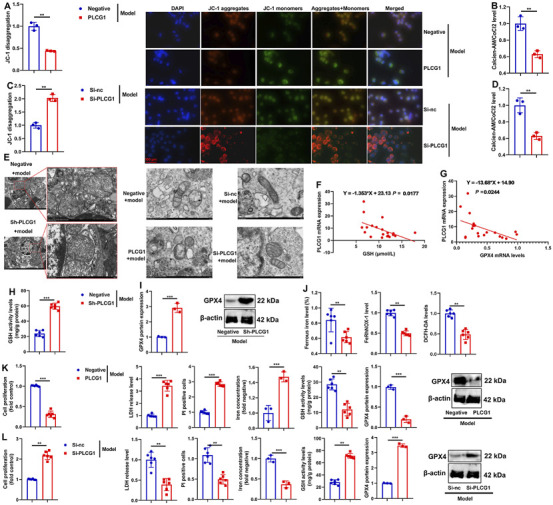
PLCG1 increased mitochondria-dependent ferroptosis in the DN model JC-1 levels **(A)** and calcein-AM/CoCl_2_ levels **(B)** in an *in vitro* model by PLCG1 plasmid; JC-1 levels **(C)** and calcein-AM/CoCl_2_ levels **(D)** in an *in vitro* model by si-PLCG1 plasmid; mitochondrial fragmentation **(E)** in kidney tissue of DN mice in an *in vitro* model by PLCG1 plasmid and in an *in vitro* model by si-PLCG1 plasmid; serum PLCG1 mRNA expression in DN patients correlated with inhibition of GSH activity levels and GPX4 mRNA expression **(F,G)**; GSH activity levels **(H)** and GPX4 protein expression **(I)**, and ferrous iron level/FeRhNOX-1/DCFH-DA **(J)** in kidney tissue of DN mice; cell growth, LDH activity, PI-positive levels, iron concentration levels, GSH activity levels, and GPX4 protein expression in high glucose-induced NRK-52E cells by PLCG1 plasmid **(βK)**; cell growth, LDH activity, PI-positive levels, and iron concentration levels, GSH activity levels and GPX4 protein expression in high glucose-induced NRK-52E cells by si-PLCG1 plasmid **(L)**. ^**^, P < 0.01; ^***^, P < 0.001.

We next investigated the role of PLCG1 in mitochondria-dependent ferroptosis using *in vitro* DN models. PLCG1 upregulation decreased both JC-1 aggregation and mitochondrial cobalt chloride (CoCl_2_) levels in high glucose-induced NRK-52E cells (Figures 6A,B), whereas PLCG1 downregulation increased these parameters (Figures 6C,D). In addition, sh-PLCG1 attenuated high glucose-induced mitochondrial fragmentation in the renal tissues of DN mice, while PLCG1 overexpression promoted mitochondrial fragmentation and siRNA-mediated PLCG1 knockdown suppressed it in NRK-52E cells (Figure 6E). These results indicate that PLCG1 exacerbates high glucose-induced mitochondrial fragmentation in DN. To assess the functional impact of PLCG1-driven mitochondrial fragmentation, we examined key ferroptosis markers. In DN patients, elevated serum PLCG1 mRNA levels correlated with reduced GSH activity and GPX4 mRNA expression (Figures 6F,G). In DN mice, sh-PLCG1 increased GSH activity and GPX4 protein expression, while decreasing ferrous iron levels, FeRhNOX-1, and DCFH-DA in renal tissue (Figures 6H–J). In high glucose-induced NRK-52E cells, PLCG1 upregulation suppressed cell growth, increased lactate dehydrogenase (LDH) activity, PI-positive cell numbers, and iron concentrations, and inhibited GSH activity and GPX4 expression (Figure 6K). Conversely, PLCG1 knockdown increased cell growth, reduced LDH release, PI positivity, and iron accumulation, and restored GSH activity and GPX4 levels (Figure 6L). Together, these findings demonstrate that PLCG1 promotes mitochondria-dependent ferroptosis in diabetic nephropathy.

### PLCG1 suppressed the LAMP2A/HSPA8 signaling pathway in the DN model

**FIGURE 7:**
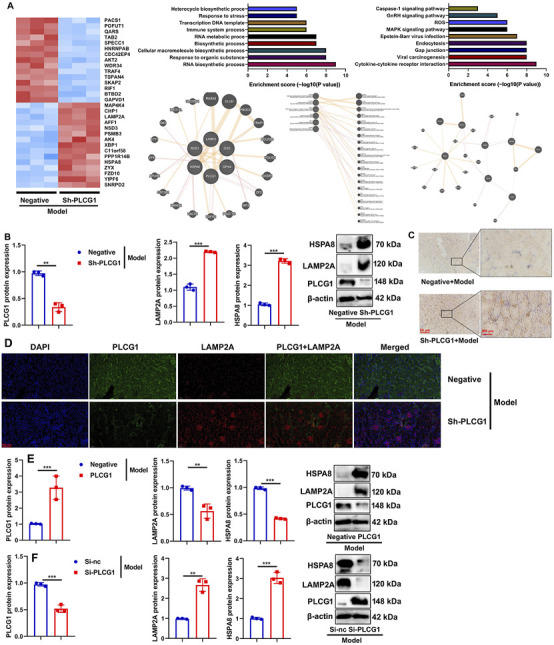
PLCG1 induced LAMP2A/HSPA8 in the DN model heat map **(A)**, PLCG1/LAMP2A/HSPA8 protein expression **(B)**, LAMP2A expression **(C)**, PLCG1+ LAMP2A expression **(D)** in kidney tissue of DN mice; PLCG1/LAMP2A/HSPA8 protein expression **(E)** in high glucose-induced NRK-52E cells by PLCG1 plasmid; PLCG1/LAMP2A/HSPA8 protein expression **(F)** in high glucose-induced NRK-52E cells by si-PLCG1 plasmid; ^**^, P < 0.01; ^***^, P < 0.001.

To investigate the mechanism by which PLCG1 regulates mitochondria-dependent ferroptosis in DN, we performed systematic analyses using both *in vivo* and *in vitro* models. In DN mice, administration of the sh-PLCG1 virus resulted in upregulated LAMP2A expression (Figure 7A), suppressed PLCG1 protein levels, and enhanced LAMP2A/HSPA8 protein expression in renal tissue (Figure 7B).

Immunohistochemical and immunofluorescence staining further confirmed that PLCG1 knockdown increased LAMP2A protein expression while reducing PLCG1 levels in the kidney tissues of DN mice (Figures 7C,D).

Corroborating these findings, PLCG1 overexpression increased its own protein expression but suppressed LAMP2A/HSPA8 levels in high glucose-induced NRK-52E cells (Figure 7E), whereas siRNA-mediated PLCG1 knockdown produced the opposite effects, reducing PLCG1 while increasing LAMP2A/HSPA8 expression (Figure 7F). Collectively, these results demonstrate that PLCG1 suppresses the LAMP2A/HSPA8 signaling pathway in diabetic nephropathy.

Collectively, these results demonstrate that PLCG1 suppresses the LAMP2A/HSPA8 signaling pathway in diabetic nephropathy.

### PLCG1 is interlinked with LAMP2A to promote LAMP2A ubiquitination

**FIGURE 8:**
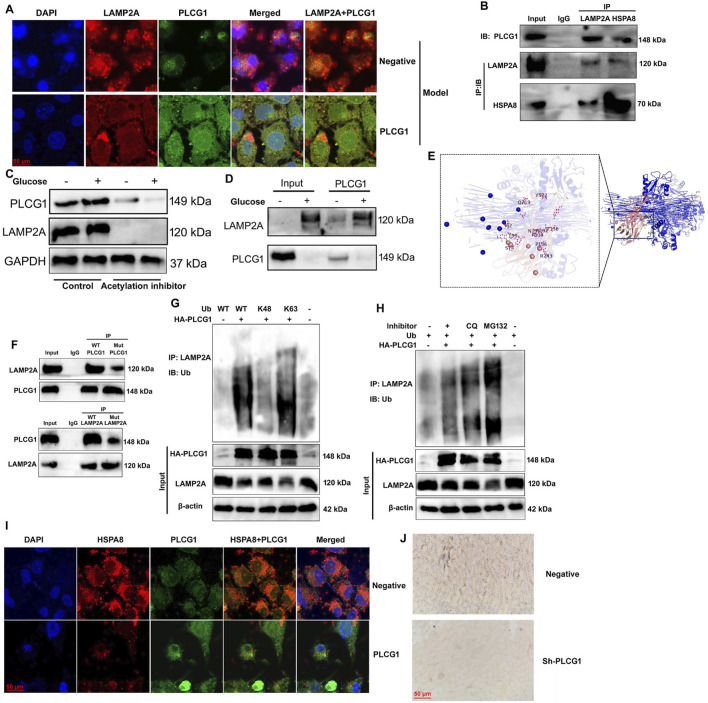
PLCG1 is interlinked with LAMP2A and promotes LAMP2A ubiquitination and PLCG1/LAMP2A expression **(A)**, PLCG1 protein was interlinked with LAMP2A and HSPA8 protein **(B)**, PLCG1 and LAMP2A protein expression **(C)**, PLCG1 protein was interlinked with LAMP2A protein **(D)** in a 3D model prediction and revealed that the LAMP2A protein interacts with the PLCG1 protein **(E)**, LAMP2A WT protein interacts with the PLCG1 WT protein **(F)**, ubiquitination of LAMP2A protein **(G,H)**. HSPA8 expression in an *in vitro* DN model (immunofluorescence, **(I)**), HSPA8 expression in the kidney tissue of DN mice (immunohistochemistry, **(J)**). Electron microscope (K).

We further elucidated the role of LAMP2A in mediating the effects of PLCG1 on mitochondria-dependent ferroptosis in a DN model. PLCG1 upregulation enhanced its own expression while suppressing LAMP2A levels in high glucose-induced NRK-52E cells (Figure 8A). Immunoprecipitation (IP) assays confirmed physical interaction among PLCG1, LAMP2A, and HSPA8 proteins (Figure 8B). High glucose stimulation increased both PLCG1 and LAMP2A protein expression, whereas the histone acetylation inhibitor trichostatin A (1.8 nM) reversed this effect (Figure 8C). Additional IP assays validated that high glucose enhances the PLCG1–LAMP2A interaction (Figure 8D).

Structural analysis through 3D modeling predicted direct interaction between LAMP2A and PLCG1 proteins (Figure 8E), which was functionally confirmed by IP studies showing wild-type LAMP2A binds wild-type PLCG1, but not mutant PLCG1, while the LAMP2A mutant fails to interact with wild-type PLCG1 (Figure 8F). Importantly, PLCG1 upregulation promoted ubiquitination of LAMP2A, specifically through K48-linked ubiquitination, under high-glucose conditions (Figures 8G,H). Then, immunofluorescence showed that PLCG1 upregulation suppressed HSPA8 expression in an *in vitro* DN model (Figure 8I). Immunohistochemistry also reported that sh-PLCG1 induced HSPA8 expression in the kidney tissue of DN mice (Figure 8J). These findings collectively demonstrate that PLCG1 interacts with LAMP2A to promote its ubiquitination in DN.

## Discussion

Diabetic nephropathy is a common complication of diabetes mellitus (Wu F. et al., 2025b), characterized by persistent proteinuria and a decreased glomerular filtration rate, ultimately leading to irreversible renal damage (Miao et al., 2025). As a major cause of end-stage renal disease (ESRD), DN progresses to ESRD in 30%–50% of affected patients worldwide (Zhang et al., 2025). Conventional management includes lifestyle and dietary modifications, along with Western medical approaches aimed at controlling blood glucose, blood pressure, blood lipids, and proteinuria to slow disease progression (Yu et al., 2025). Nevertheless, a significant number of patients experience disease deterioration and eventually advance to ESRD. Interestingly, our experiment revealed that PLCG1 expression was upregulated in DN patients or DN mice. Single-cell analysis of PLCG1 expression levels in renal cells of the DN model showed that high glucose induced PLCG1 histone acetylation in the DN model. Safari-Alighiarloo et al. (2017) identified that PLCG1 is a key marker in type 1 diabetes. These results collectively indicate that high glucose promotes histone acetylation of PLCG1 and upregulates its expression in renal cells during diabetic nephropathy.

Ferroptosis, a form of programmed cell death, serves as a key driver in the onset and progression of DN and is closely associated with damage to intrinsic renal cells under diabetic conditions (Chen et al., 2025). Traditional Chinese medicine can improve DN by regulating the ferroptosis of intrinsic renal cells, and it has good research and application prospects (Shan et al., 2025). Clinical studies have shown that serum ferritin levels are significantly elevated in DN patients, while kidney biopsy specimens exhibit decreased GPX4 expression and accumulation of lipid peroxides, further supporting a strong link between DN and ferroptosis (Mu et al., 2025). Defined as an iron-dependent regulated cell death process, ferroptosis is primarily characterized by iron-driven reactive oxygen species accumulation and depletion of polyunsaturated fatty acids in the plasma membrane (Shan et al., 2025). The regulatory mechanisms of ferroptosis are complex, involving iron metabolism, lipid and amino acid metabolism pathways, and signaling pathways mediated by coenzyme Q, P53, guanosine triphosphate cyclohydrolase 1, and mitochondrial voltage-dependent anion channels (Shen et al., 2025). Morphologically, cellular ferroptosis presents distinct features such as marked shrinkage or swelling of mitochondria, increased membrane density, and reduction or loss of mitochondrial cristae (Ou and Zhang, 2025). In the present study, we found that sh-PLCG1 alleviated DN and oxidative stress in a mouse model, whereas PLCG1 enhanced mitochondria-dependent ferroptosis in DN. He et al. reported elevated PLCG1 in diabetes mellitus rats and high glucose-treated HRECs (He et al., 2022), but their study did not focus on diabetic kidney involvement. Our research further reveals that PLCG1 promotes high glucose-induced mitochondrial oxidative stress and mitochondrial injury in renal cells, exacerbates cellular ferroptosis, and accelerates the progression of DN.

Lysosome-associated membrane protein 2A (LAMP2A) serves as the sole rate-limiting component of the chaperone-mediated autophagy (CMA) pathway (Issa et al., 2018). In CMA, substrates are recognized and delivered to the lysosomal receptor LAMP2A, then translocated into the lysosomal lumen with the assistance of the resident chaperone heat shock protein 90 (HSP90) (Ferreira et al., 2022). In most cancer cell lines, CMA activity is markedly upregulated, accompanied by abnormally elevated LAMP2A levels. Studies have revealed that the highly activated CMA system in tumor cells primarily facilitates tumor growth, proliferation, and invasion through aerobic glycolysis; promotes degradation of tumor suppressor proteins such as Rho-related GTP-binding protein RhoE (RND3) and non-phosphorylated PED (phosphoprotein enriched in diabetes); and enhances tumor cell survival, proliferation, metastasis, and drug resistance, thereby exerting an oncogenic role (Lee et al., 2022; Li et al., 2024; Pajares et al., 2018). In this study, we demonstrated that PLCG1 upregulation promotes K48-linked ubiquitination of the LAMP2A protein in high glucose-induced NRK-52E cells. Cheng et al. indicated that PLCG1 is regulated by defects in chaperone-mediated autophagy involving LAMP2A (Cheng et al., 2025). Our findings reveal for the first time that PLCG1 can regulate LAMP2A in a diabetic kidney model. Although we have identified a role for this pathway in ferroptosis, the specific downstream mechanisms—whether it regulates ferroptosis, autophagy, or other cellular functions—warrant further investigation. In our subsequent experiments, we will explore the tyrosine phosphorylation of DN in mesangial cells and compare it with the conclusions of this study. This will enhance our understanding of DN. Emerging evidence suggests a connection between ferroptosis and endoplasmic reticulum (ER) stress, while HSPA8 has been shown to promote cell survival under ER stress conditions (Miao et al., 2023; Stricher et al., 2013). The ferroptosis inhibitor Fer-1 can reverse apoptosis induced by HSPA8 knockdown and simultaneously suppress the upregulation of intracellular ROS and LDH levels. As a key regulator in DN, GPX4 is directly bound and positively regulated by HSPA8, which acts as its upstream molecular partner (Zhou et al., 2022). Under pathological conditions, suppressed HSPA8 expression leads to decreased GPX4 activity, thereby promoting iron overload in DN renal cells (Deng et al., 2023). The present study provides the first evidence that PLCG1 suppressed the LAMP2A/HSPA8 signaling pathway in a DN model. Ikami et al. (2022) demonstrated that LAMP2A mediates the interaction between the lysosomal cytoplasmic surface and HSPA8. This study found that PLCG1 suppresses LAMP2A/HSPA8 to promote mitochondrial oxidative stress, thereby increasing cellular ferroptosis and exacerbating the progression of DN. However, whether LAMP2A represents the exclusive downstream target of PLCG1 requires further investigation.

**FIGURE 9:**
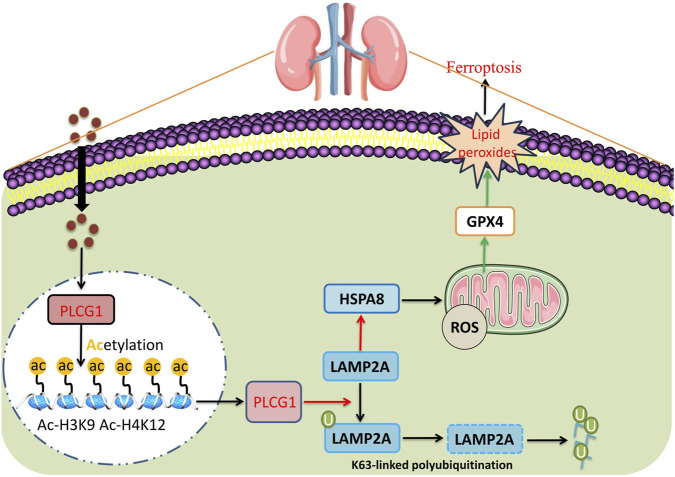
High glucose-induced PLCG1 histone acetylation to promote ferroptosis by LAMP2A/HSPA8 in a diabetic nephropathy model.

Our study identifies a novel mechanism by which PLCG1 exacerbates DN progression: through inhibition of the LAMP2A/HSPA8 pathway, it promotes mitochondrial oxidative stress and increases cellular ferroptosis (Figure 9). Specifically, PLCG1 upregulation facilitates K48-linked ubiquitination of LAMP2A protein in high glucose-induced NRK-52E cells (Figure 9). Importantly, PLCG1 represents not only a promising clinical biomarker for early DN detection but also a potential therapeutic target. By screening for compounds that inhibit PLCG1 activity, it may be possible to mitigate glucose-induced ferroptosis, thereby offering new strategies for treating DN and other diabetic complications.

## Data Availability

The raw data supporting the conclusions of this article will be made available by the authors, without undue reservation.
